# Combination Therapy of Half-Dose Resmetirom and Metformin Attenuates Metabolic Dysfunction-Associated Steatohepatitis Through Improving Cholesterol Metabolism and Inflammation

**DOI:** 10.3390/biomedicines13061315

**Published:** 2025-05-27

**Authors:** Wenxiu Liu, Fan Yao, Jinghan Wang, Nan Shao, Xinxin Cao, Zhengqi Dong, Bin Zhang, Xiaobo Sun

**Affiliations:** 1 Institute of Medicinal Plant Development, Peking Union Medical College and Chinese Academy of Medical Sciences, Beijing 100193, China; liuwenxiu1sdu@163.com (W.L.); fany_strive@163.com (F.Y.); andreas777@foxmail.com (J.W.); 19104354417@163.com (N.S.); xinxincao97@163.com (X.C.); zqdong@implad.ac.cn (Z.D.); 2 Key Laboratory of Bioactive Substances and Resources Utilization of Chinese Herbal Medicine, Ministry of Education, Beijing 100193, China; 3 Diabetes Research Center, Chinese Academy of Medical Sciences, Beijing 100193, China; 4 Key Laboratory of Efficacy Evaluation of Chinese Medicine Against Glyeolipid Metabolism Disorder Disease, State Administration of Traditional Chinese Medicine, Beijing 100193, China

**Keywords:** metabolic dysfunction-associated steatohepatitis (MASH), combination therapy, metformin, resmetirom, lipidomic analysis, transcriptomic analysis, cholesterol metabolism

## Abstract

**Background/Objectives**: Metabolic dysfunction-associated steatohepatitis (MASH) has become the leading cause of hepatocellular carcinoma and liver disorders globally. Nevertheless, only one expensive drug, resmetirom (Res), has been approved by the FDA for MASH treatment to date. However, its high price has imposed a heavy financial burden on patients. **Methods**: In this study, half-dose Res and low-dose metformin (Met) (referred to as RM) were administered in combination to treat MASH models in vitro and in vivo. We utilized transcriptome and lipidomics sequencing to assess the efficacy of RM in improving MASH. Our goal was to systemically compare the therapeutic effects of RM, Met, and Res on MASH and elucidate the underlying mechanisms. **Results**: Our results demonstrated that RM was comparable to Res and superior to Met in reducing lipid production in vitro, attenuating lipid accumulation, inhibiting inflammation, and improving fibrosis in vivo. Transcriptome and lipidome analyses further revealed that RM regulated the expression of genes and lipids in a manner similar to Res, particularly in pathways related to cholesterol metabolism and inflammation. Further validation showed that RM facilitated cholesterol transformation by robustly promoting the expression of CYP7A1, thereby mitigating MASH. **Conclusions**: Collectively, our findings highlight that a combination of half-dose Res and Met is equivalent to Res alone in terms of MASH treatment efficacy. This study provides a novel therapeutic strategy that is not only effective for MASH treatment but also reduces the economic burden on patients.

## 1. Introduction

Metabolic dysfunction-associated steatotic liver disease (MASLD, previously known as NAFLD) is a common liver disorder that affects approximately 30% of the global population, with an increasing incidence [1]. Given its complex pathogenesis, MASLD is also closely related to obesity, lipodystrophy, and type 2 diabetes mellitus (T2DM), severely threatening human health [2]. MASLD is a progressive disease, and metabolic dysfunction-associated steatohepatitis (MASH, previously known as NASH) represents a more advanced stage [3]. MASH is characterized by lipid accumulation, inflammation, and fibrosis in the liver [4]. Mechanistically, excessive lipid deposition in hepatocytes contributes to lipid metabolism disorder, which includes increased lipid uptake and biosynthesis, as well as decreased lipid degradation [5]. Free fatty acids (FFA) are lipotoxic to hepatocytes, causing inflammation and necrosis of hepatocytes while also activating macrophages and hepatic stellate cells. This activation leads to liver inflammation and fibrosis. As the disease progresses, MASH can further develop into cirrhosis and hepatocellular carcinoma (HCC) [6].

Although MASH has attracted widespread attention due to its high prevalence and potential progression, few effective therapies have been approved. Resmetirom (Res), a thyroid hormone receptor-beta (THR-β) agonist, was approved by the FDA in March 2024 and became the only listed drug for MASH treatment [7,8]. The recommended clinical dose of Res is 80 mg/kg or 100 mg/kg; however, its high cost, USD 47,400 per year, limits its accessibility for MASH patients [9,10]. Additionally, MASH is a chronic disease, and patients need to take long-term medication, which may increase the risk of adverse reactions [11]. Based on the WHO-VigiAccess database (https://www.vigiaccess.org (accessed on 19 May 2025)), the most common types of adverse reactions to resmetirom were gastrointestinal disorders, with 182 cases reported (21%). Additionally, nausea was identified as a common adverse reaction to resmetirom, which was related to gastrointestinal disorders [12]. Currently, other drugs used in metabolic disease therapies, including hypoglycemic agents, can also be employed to assist MASH treatment [13,14]. It is noteworthy that T2DM is bidirectionally associated with MASLD [15]. It was estimated that 55.5% of T2DM patients have MASLD [16], while the prevalence of T2DM in MASLD and MASH patients is 22.5% and 43.63% [17]. Moreover, T2DM accelerates the progression of liver fibrosis and increases the risk of HCC in patients with MASLD [18]. Metformin (Met), the first-line drug for treating T2DM, has been shown to have positive effects in MASLD treatment in numerous studies [19]. However, long-term use of Met may lead to potential side effects due to its high clinical oral dose, such as gastrointestinal symptoms [20]. Therefore, to reduce the required dose and treatment cost of Res, we proposed and evaluated the combined effect of a half-dose of Res and Met in the MASH model and explored its underlying molecular mechanisms.

A plethora of evidence indicates the critical role of cholesterol homeostasis disorders in the pathogenesis of MASH, as they can induce oxidative stress and inflammation. Hepatocytes obtain cholesterol mainly through endogenous synthesis (with the rate-limiting enzyme being 3-hydroxy-3-methylglutaryl coenzyme A reductase (HMGCR)) and exogenous uptake [21]. Hepatocytes uptake low-density lipoprotein particles and chylomicron remnants via low-density lipoprotein receptor (LDLR)-mediated endocytosis, which helps to remove cholesterol from the circulation and maintain normal cholesterol levels in the plasma [22]. The primary way to clear cholesterol in hepatocytes is to convert it into bile acids, with cholesterol 7 alpha-hydroxylase (cytochrome P450 family 7 subfamily A member 1, CYP7A1) and sterol-27-hydroxylase (cytochrome P450 family 27 subfamily A member 1, CYP27A1) serving as the rate-limiting enzymes [23,24]. Subsequently, ATP-binding cassette subfamily G member 5 or G member 8 (ABCG5/G8) excretes bile acids into the bile [24]. Therefore, intervening in these pathways to counteract liver lipid accumulation is a potential strategy to relieve MASH.

In this study, we evaluated the protective effects of the combination of Res and Met at a halved clinical dose, both in vitro and in vivo. RM had the same effect as Res and Met on ameliorating lipid deposition and liver fibrosis, but its role in improving inflammation was better than that of Res and Met in db/db mice. Our experimental data elucidated that RM mitigated MASH comparably to Res, while better than Met. These findings confirmed that the combination of a halved clinical dose of Res and Met can be used as an innovative and effective therapy for MASH treatment, which lowers the huge economic burden on patients caused by clinical-dose Res.

## 2. Materials and Methods

### 2.1. Animals

Male C57BL/6J and db/db mice (6-week-old) were purchased from Cavens Experimental Animal Co., Ltd. (Changzhou, China). All mice were fed under a 12-h light–dark cycle at a constant temperature (22 ± 2 °C) and humidity (56 ± 5%), with sufficient normal diet and water. All animal experiments were approved by the Ethical Committee on Animal Experimentation of the Chinese Academy of Medical Sciences and Peking Union Medical College (ethical code: SLXD-20240430010). After one week of adaptation, mice were randomly divided into the control group, model group, Met treatment group (Metformin, Bristol-Myers Squibb (China) (Shanghai, China), 205 mg/kg and 410 mg/kg, i.g.), Res treatment group (Resmetirom, Shanghai Yuanye Bio-Technology Co., Ltd., Shanghai, China, 16.4 mg/kg, i.g.), and RM treatment group (metformin 205 mg/kg + resmetirom 8.2 mg/kg, i.g.). After nine weeks of administration, all mice were sacrificed. Serum was collected, and livers were frozen in liquid nitrogen and fixed in 4% paraformaldehyde.

### 2.2. Cell Culture and Treatment

The HepG2 cell line was purchased from Pricella Biosciences (Suzhou, China) and AML12 cells were obtained from the Cell Bank/Stem Cell Bank, Chinese Academy of Sciences. Both cell lines were cultured in DMEM (GIBCO BRL, Grand Island, NY, USA) supplemented with 10% FBS (GIBCO BRL, Grand Island, NY, USA) and 1% ampicillin/streptomycin (GIBCO BRL, Grand Island, NY, USA) in an incubator at 37 °C with 5% CO_2_. HepG2 cells were seeded at a density of 1 × 10^4^ cells/well in 96-well plates (or 6 × 10^5^ cells/well in 6-well plates) and allowed to adhere for 24 h prior to treatment. AML12 cells were seeded at a density of 5 × 10^3^ cells/well in 96-well plates (or 3 × 10^5^ cells/well in 6-well plates) and allowed to adhere for 24 h prior to treatment. Subsequently, the cells were exposed to FFA (0, 0.25, 0.5, 1, 1.5, and 2 mM) composed of oleic acid (Sigma-Aldrich, St. Louis, MO, USA) and palmitic acid (Sigma-Aldrich, St. Louis, MO, USA) (2:1 ratio) for 24 h. Cell viability and TG content were detected using a cell counting kit-8 (CCK8) kit (GLPBIO, Montclair, CA, USA) and a TG assay kit (Nanjing Jiancheng Biotechnology Research Institute Co., Ltd., Nanjing, China), respectively.

To detect therapeutic effects, HepG2 and AML12 cells were seeded in 6-well plates and cultured for 24 h. Then, HepG2 cells were treated with 1 mM FFA, and AML12 cells were treated with 1.5 mM FFA, both in the presence or absence of drugs (50 μM Res, 10 μM Met, 40 μM Met, 25 μM Res + 10 μM Met (RM)) for 24 h. After treatment, cells were collected for RNA extraction or for detection of TG and TC content using the TG and TC assay kit (Nanjing Jiancheng Biotechnology Research Institute Co., Ltd., Nanjing, China) according to the manufacturer’s instructions. For Oil Red O and Bodipy staining, HepG2 and AML12 cells were seeded at a density of 2 × 10^5^ and 1 × 10^5^ cells/well, respectively, on glass coverslips in 12-well plates and cultured for 24 h. The cells were then treated as described above. After that, the cells were fixed with 4% paraformaldehyde (Solarbio, Beijing, China) and stained with the corresponding reagents according to the Oil Red O assay kit (Beyotime Biotechnology Co., Ltd., Shanghai, China) or Bodipy reagent (Invitrogen, Carlsbad, CA, USA) protocols. The nuclei were stained with hematoxylin (Beyotime Biotechnology Co., Ltd., Shanghai, China) or DAPI (Zhongshan Golden Bridge Biological Technology Co., Ltd., Beijing, China), respectively. The stained cells were visualized by TissueGnostics (Vienna, Austria).

### 2.3. Real-Time Quantitative Polymerase Chain Reaction (RT-qPCR)

Total RNA was extracted from HepG2 cells, AML12 cells, and liver tissues using Trizol reagent (Takara Bio, Shiga, Japan). After the RNA concentration was measured by NanoDrop (Thermo Fisher Scientific, Waltham, MA, USA), cDNA was synthesized through reverse transcriptional reaction using a reverse transcriptase kit (Takara Bio, Shiga, Japan). Then, RT-qPCR was performed via fluorescence quantification kits (Takara Bio, Shiga, Japan). All primer sequences are displayed in Table 1.

### 2.4. Blood Biochemical Parameters

The levels of triglyceride (TG), total cholesterol (TC), alanine aminotransferase (ALT), and aspartate aminotransferase (AST) in mouse serum were detected using the Beckman Coulter AU480 automatic biochemical analyzer according to the manufacturer’s protocols (Biosano, Beijing, China). The levels of tumor necrosis factor-alpha (TNF-α) and interleukin (IL)-10 in serum were measured with enzyme-linked immunosorbent assay (ELISA) kits (Elabscience, Wuhan, China) according to the instructions provided.

### 2.5. Histopathology

After the liver tissues were fully fixed with 4% paraformaldehyde, they were embedded in paraffin to make slices. Liver sections of every sample were stained with hematoxylin and eosin (H&E), Sirius red, and Masson reagents. Subsequently, the histological changes were assessed with a microscope (LeicaFS1000, Wetzlar, Germany).

For Oil Red O staining, the fixed liver tissues were rinsed and then embedded with OCT to make slices. Liver sections of every sample were stained with Oil Red O reagents. Subsequently, the histological features of liver tissues were observed with the microscope (LeicaFS1000, Wetzlar, Germany).

### 2.6. Transmission Electron Microscope (TEM)

After mice sacrifice, the liver tissues were immersed in electron microscope liquid, and the following operation was the same as previously described [25]. Ultimately, the ultrastructure of liver tissues was observed by TEM (Hitachi, Tokyo, Japan).

### 2.7. Protein Extraction and Western Blot

Proteins in liver tissues were extracted using RIPA lysis buffer (CW2333S, CWBIO) with protease inhibitors (CW2200S, CWBIO). Protein samples were separated using sodium dodecyl-sulfate polyacrylamide gel (SDS-PAGE) electrophoresis and transferred onto an NC membrane (Cytiva, Uppsala, Sweden). Then, the membrane was blocked at room temperature for 2 h and incubated with anti-CYP7A1 (18054-1-AP, Proteintech, Wuhan, China), anti-ABCG5 (27722-1-AP, Proteintech, Wuhan, China), anti-LDLR (10785-1-AP, Proteintech, Wuhan, China), and anti-β-ACTIN (AC026, ABclonal Technology, Wuhan, China) primary antibodies overnight at 4 °C. Subsequently, membranes were incubated with corresponding secondary antibodies and imaged using a Touch Imager System (e-BLOT, Shanghai, China). Finally, ImageJ 1.53k software (National Institutes of Health, Bethesda, MD, USA) was applied to analyze the protein expression, and β-ACTIN was used as the control.

### 2.8. Transcriptomic Sequencing Analysis

After total RNA extraction of mice liver tissues, the integrity and total amount of RNA were examined accurately by Agilent 2100 bioanalyzer (Agilent Technologies, Santa Clara, CA, USA). Then, mRNA was purified from total RNA by using poly-T oligo-attached magnetic beads for library preparation.

After the library was qualified, different libraries were pooled together for Illumina sequencing according to the effective concentration and the amount of target offline data. All raw reads were processed by fastp software (version 0.19.7), and all downstream analysis was based on these high-quality clean data. Next, differential expression analysis, GO and KEGG enrichment analysis of DEGs, GSEA, and other analyses were all performed.

### 2.9. Lipidomics Sequencing Analysis

Precooled methanol and MTBE were added to 100 mg of liver tissue powder and incubated. Subsequently, MS-grade water was added to the mix, and the upper organic phase was collected by centrifugation. The solvent mixture was added to the lower layer to re-extract and collect the upper organic phase. The twice collected organic phase was concentrated and redissolved with isopropanol and then analyzed by the LC-MS/MS system. UHPLC-MS/MS analyses were performed using a Vanquish UHPLC system (Thermo Fisher, Dreieich, Germany) coupled with an Orbitrap Q Exactive^TM^ HF mass spectrometer (Thermo Fisher, Dreieich, Germany). Samples were injected into a Thermo Accucore C30 column (150 × 2.1 mm, 2.6 μm) at a flow rate of 0.35 mL/min. The column temperature was 40 °C. Mobile phase buffer A was acetonitrile/water (6/4) with 10 mM ammonium acetate and 0.1% formic acid, whereas buffer B was acetonitrile/isopropanol (1/9) with 10 mM ammonium acetate and 0.1% formic acid. The Q Exactive^TM^ HF mass spectrometer was operated in positive and negative polarity modes to further analyze samples.

The raw data files generated by UHPLC-MS/MS were processed using Lipidsearch to perform peak alignment, peak picking, and quantitation for each metabolite. Ultimately, qualitative and quantitative information about the substances in different samples was obtained. Further data processing and analysis were performed by using the R language (version R-3.4.3) and corresponding R packages (version R-3.4.3).

### 2.10. Statistical Analysis

Statistical analysis was performed by the software GraphPad Prism 8.0. All quantitative data were assessed for normality using the Shapiro-Wilk test. The homogeneity of variances was verified using Bartlett’s test for parametric data. Datasets satisfying both normality and equal variance were analyzed with parametric tests. Comparisons of multiple groups were determined by one-way ANOVA with Tukey’s post hoc. All results were represented as means ± SD, and *p* < 0.05 was considered statistically significant.

## 3. Results

### 3.1. A Combination of Half-Dose Res and Met Alleviated Lipid Metabolism Disorders in HepG2 Cells Treated with FFA

**Figure 1:**
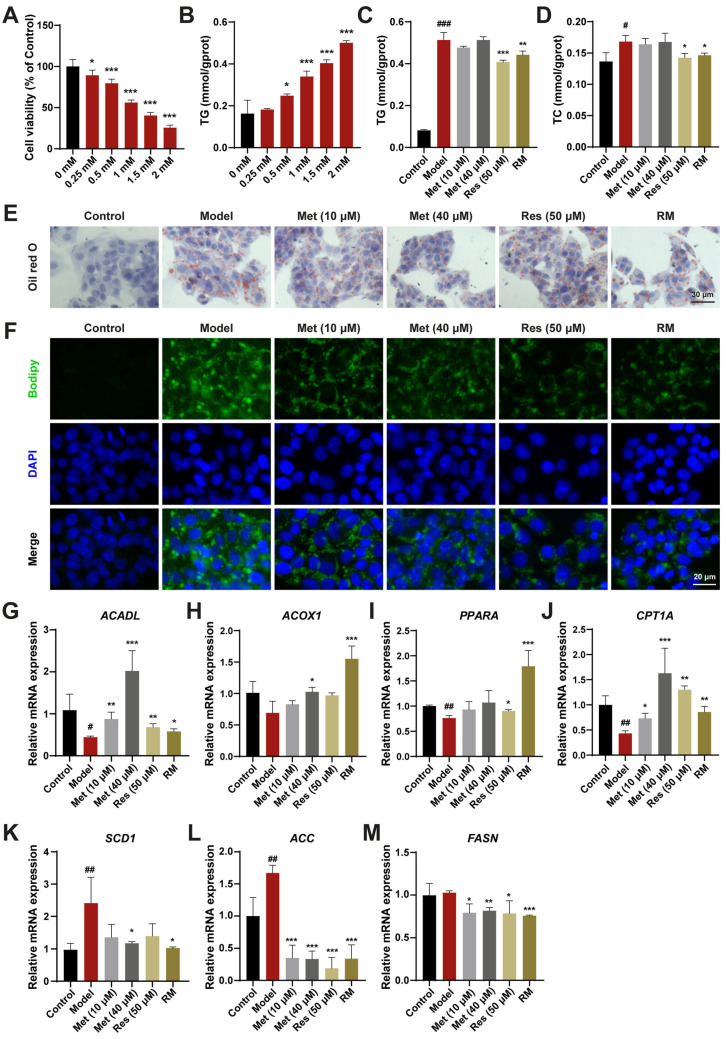
RM ameliorated lipid homeostasis in HepG2 cells treated with FFA. (**A**) Effects of FFA (0, 0.25, 0.5, 1, 1.5, and 2 mM) on HepG2 cell viability (*n* = 6). (**B**) Effects of FFA (0, 0.25, 0.5, 1, 1.5, and 2 mM) on HepG2 cell TG levels (*n* = 3). (**C**,**D**) The levels of TG and TC in HepG2 cells (*n* = 3). (**E**,**F**) Representative images of Oil Red O and Bodipy staining of HepG2 cells (*n* = 3, scale bar: 30 μm and 20 μm). (**G**–**M**) The mRNA levels of HepG2 cells were examined by RT-qPCR (*n* = 3). Data represent mean ± SD; # *p* < 0.05, ## *p* < 0.01, ### *p* < 0.001 versus the control group; * *p* < 0.05, ** *p* < 0.01, and *** *p* < 0.001 versus the model group.

To assess the curative effects of Res and Met on MASH, a HepG2 cell model treated with FFA was established. Upon treatment with 1 mM FFA, HepG2 cells exhibited significantly reduced cell viability and a marked increase in TG content (Figure 1A,B). The lipid-lowering effects of RM and Res were confirmed by measuring TG and TC contents in FFA-induced cells, whereas these effects were not significant in the low- and high-dose Met groups (Figure 1C,D). Oil Red O and Bodipy staining results further demonstrated that RM clearly decreased cellular lipid accumulation in FFA-treated cells. The RM group had fewer Oil Red O areas and lower green fluorescence intensity than the Met (10 μM) group and was equivalent to the Res and Met (40 μM) groups (Figure 1E,F).

To elucidate the mechanisms underlying the reduction of lipid accumulation by these drugs, we evaluated the expression level of genes involved in fatty acid oxidation and lipogenesis. Compared with the model group, HepG2 cells treated with Met (10 μM and 40 μM), Res, and RM exhibited increased mRNA expression of genes modulating fatty acid degradation, including long-chain acyl-coenzyme A dehydrogenase (*ACADL*), acyl-coenzyme A oxidase 1 (*ACOX1*), proliferator-activated receptor α (*PPARA*), and carnitine palmitoyltransferase 1α (*CPT1A*). Conversely, the expression of genes regulating fatty acid synthesis, such as stearoyl-CoA desaturase 1 (*SCD1*), acetyl-CoA carboxylase (*ACC*), and fatty acid synthase (*FASN*), was decreased (Figure 1G–M). Interestingly, Met enhanced the expression of lipid metabolism-related genes in a dose-dependent manner, and RM achieved the same efficacy as high-dose Res or Met alone (Figure 1G–M). These results suggest that RM effectively protects HepG2 cells from lipid metabolism disorders induced by FFA.

### 3.2. A Combination of Half-Dose Res and Met Decreased Lipid Accumulation in AML12 Cells Treated with FFA

**Figure 2:**
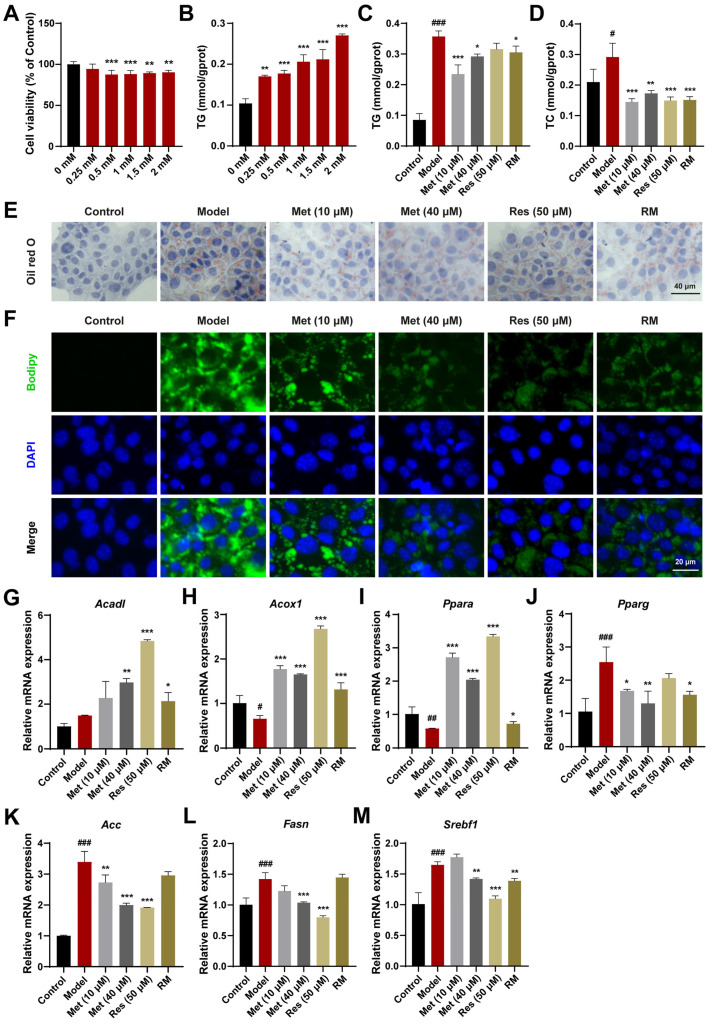
RM improved lipid metabolism in AML12 cells treated with FFA. (**A**) Effects of FFA (0, 0.25, 0.5, 1, 1.5, and 2 mM) on AML12 cell viability (*n* = 6). (**B**) Effects of FFA (0, 0.25, 0.5, 1, 1.5, and 2 mM) on AML12 cell TG levels (*n* = 3). (**C**,**D**) The levels of TG and TC in AML12 cells (*n* = 3). (**E**,**F**) Representative images of Oil Red O and Bodipy staining of AML12 cells (*n* = 3, scale bar: 40 μm and 20 μm). (**G**–**M**) The mRNA levels of AML12 cells were examined by RT-qPCR (*n* = 3). Data represent mean ± SD; # *p* < 0.05, ## *p* < 0.01, ### *p* < 0.001 versus the control group; * *p* < 0.05, ** *p* < 0.01, and *** *p* < 0.001 versus the model group.

AML12 cells, a mouse hepatocyte cell line, were also used to validate the therapeutic effects. Based on the results of CCK8 and TG assays, a concentration of 1.5 mM FFA was selected for subsequent cell experiments (Figure 2A,B). Met (10 μM and 40 μM), Res, and RM reduced TC and TG levels in AML12 cells treated with FFA (Figure 2C,D). The reduction in lipid accumulation was further confirmed by Oil Red O and Bodipy staining. With RM treatment, AML12 cells displayed fewer Oil Red O areas and less green fluorescence intensity in comparison with the model group (Figure 2E,F). Of note is that the RM group showed fewer Oil Red O areas and green fluorescence intensity compared to the low-dose Met group and was comparable to the high-dose Met and Res groups in that (Figure 2E,F).

In addition, to elucidate the mechanism of lipid reduction, we evaluated the expression levels of genes regulating fatty acid oxidation and lipogenesis in AML12 cells. Compared with the model group, Met (10 μM and 40 μM), Res, and RM effectively upregulated the expression of genes modulating fatty acid degradation (*Acadl*, *Acox1*, and *Ppara*) and downregulated the expression of genes regulating fatty acid synthesis (proliferator-activated receptor γ (*Pparg*), *Acc*, *Fasn*, and sterol regulatory element binding transcription factor 1 (*Srebf1*)) in AML12 cells (Figure 2G–M). These results indicate that RM effectively protects AML12 cells against lipid accumulation induced by FFA and exhibits comparable therapeutic effects to Res.

### 3.3. Concomitant Half-Dose Res and Met Ameliorated Hepatic Lipid Accumulation and Metabolism Disorders in db/db Mice

**Figure 3:**
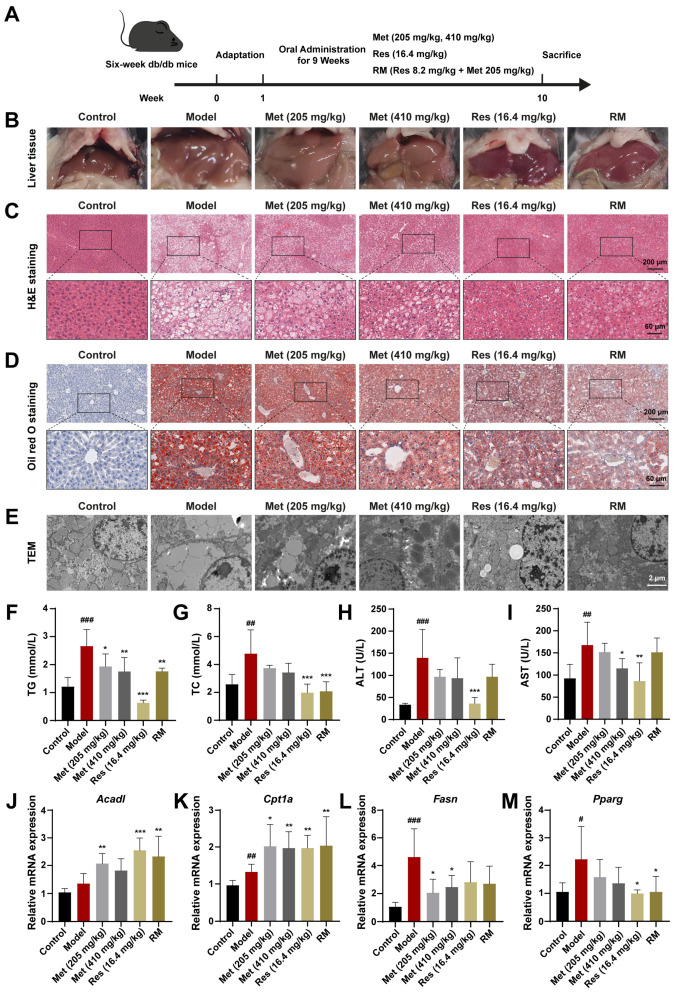
Met, Res, and RM improved systemic lipid homeostasis in db/db mice (*n* = 6/group). (**A**) Procedure for the administration of Met, Res, and RM. (**B**–**E**) Representative images of the liver specimen, H&E staining (scale bars: 200 µm and 60 µm; magnification: 20× and 63×), Oil Red O staining (scale bars: 200 µm and 60 µm; magnification: 20× and 63×), and TEM (scale bars: 2 µm; magnification: 6000×). (**F**) Serum TG levels. (**G**) Serum TC levels. (**H**) Serum ALT levels. (**I**) Serum AST levels. (**J**–**M**) The mRNA levels of liver tissues were examined by RT-qPCR. Data represent mean ± SD; # *p* < 0.05, ## *p* < 0.01, and ### *p* < 0.001 versus the control group; * *p* < 0.05, ** *p* < 0.01, and *** *p* < 0.001 versus the model group.

To determine the therapeutic effects of RM on MASH progression in vivo, we treated db/db mice with RM, Res, and low and high doses of Met via oral gavage for 9 weeks (Figure 3A). Macroscopical changes were observed in liver tissues among these groups (Figure 3B). Compared with the control group, the livers of untreated db/db mice featured hepatomegaly, passivated liver edge, yellow coloration, and a greasy appearance. In contrast, Res, Met, and RM attenuated these alterations (Figure 3B). Histopathological results of H&E and Oil Red O staining showed balloon-like changes, inflammatory cell infiltration, and excessive lipid droplet accumulation in the livers of db/db mice (Figure 3C,D). TEM of liver ultrastructure in the model group showed increased large lipid droplets and broken mitochondria (Figure 3E). However, Met administration improved these histological and microscopic changes in a dose-dependent manner, and RM equivalently ameliorated these pathological differences compared to Res (Figure 3C,E).

Additionally, several indices related to systemic lipid metabolism and liver function were detected. Compared to the control group, serum levels of TG, TC, ALT, and AST were prominently elevated in the model group (Figure 3F–I). Administration of Res and RM significantly reduced both TG and TC levels, but the effect of Met on improving TC was not as good as that of TG (Figure 3F,G). Meanwhile, although Met (205 and 410 mg/kg) and RM clearly led to decreased ALT and AST levels, these reductions were not as significant as those observed with Res (Figure 3H,I).

Moreover, the results of RT-qPCR revealed that the expression of *Acad1* and *Cpt1a* was elevated by more than 1.5-fold vs. control, while the expression of *Fasn* and *Pparg* was downregulated following treatment with Met, Res, and RM (Figure 3J–M). These findings suggest that these medications remedy liver lipid metabolism disorders in db/db mice. Collectively, RM supplementation obviously mitigated lipid metabolism disorders and liver function deterioration in MASH mice.

### 3.4. Combination Therapies Improved Inflammation and Liver Fibrosis in db/db Mice

**Figure 4:**
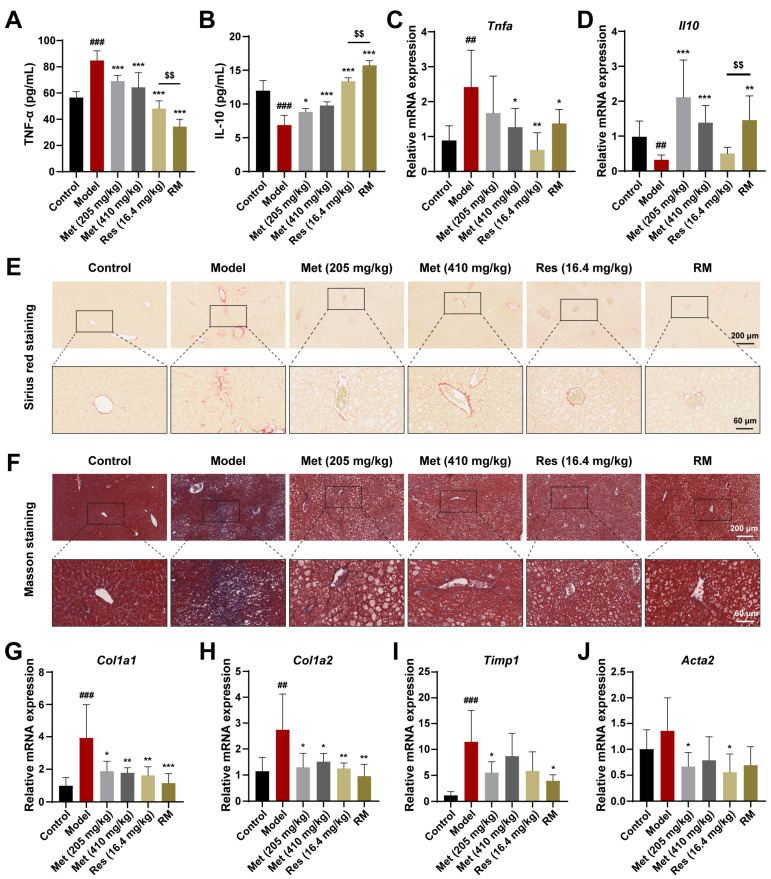
Met, Res, and RM improved inflammation and liver fibrosis in db/db mice (*n* = 6/group). (**A**) Serum TNF-α levels by ELISA. (**B**) Serum IL-10 levels by ELISA. (**C**,**D**) The mRNA levels of liver tissues were examined by RT-qPCR. (**E**,**F**) Representative images of Sirius red staining (scale bars: 200 µm and 60 µm; magnification: 20× and 63×) and Masson staining (scale bars: 200 µm and 60 µm; magnification: 20× and 63×). (**G**–**J**) The mRNA levels of liver tissues were examined by RT-qPCR. Data represent mean ± SD; ## *p* < 0.01, ### *p* < 0.001 versus the control group; * *p* < 0.05, ** *p* < 0.01, and *** *p* < 0.001 versus the model group; $$ *p* < 0.01 versus Res (16.4 mg/kg) group.

To further confirm whether Met, Res, and RM could attenuate inflammation in vivo, we detected serum inflammatory factors using ELISA kits. In the model mice, TNF-α levels were significantly increased, while IL-10 levels were significantly decreased; however, these changes were reversed in all four treatment groups (Figure 4A,B). Interestingly, Met blunted the inflammation response in a dose-dependent manner, and RM was more effective than Res in this regard (Figure 4A,B). Meanwhile, the mRNA levels of TNF-α and IL-10 in liver tissues mirrored the ELISA data (Figure 4C,D).

Next, to investigate the fibrotic changes in MASH progression, we performed Sirius red and Masson staining on liver tissues from db/db mice receiving or not receiving drug treatment. The results unveiled that the fibrosis was aggravated in the model mice, but this phenomenon was improved by Met, Res, and RM treatments (Figure 4E,F). Similarly, the mRNA levels of profibrotic markers (collagen type I alpha 1 (*Col1a1*), collagen type I alpha 2 (*Col1a2*), tissue inhibitor of metal protease 1 (*Timp1*), and alpha actin 2 (*Acta2*)) were obviously restored in the livers of db/db mice after administrating Met, Res, and RM (Figure 4G–J). Intriguingly, RM was as effective as Res and high-dose Met (410 mg/kg) in ameliorating liver fibrosis (Figure 4G–J). These data reveal that RM notably inhibits both inflammation and hepatic fibrosis in the MASH model. Importantly, RM was more effective in reducing inflammation than either Res or high-dose Met alone.

### 3.5. Resmetirom and Metformin Extensively Regulated the Expression of Genes and Multiple Metabolism Processes in the Liver

**Figure 5:**
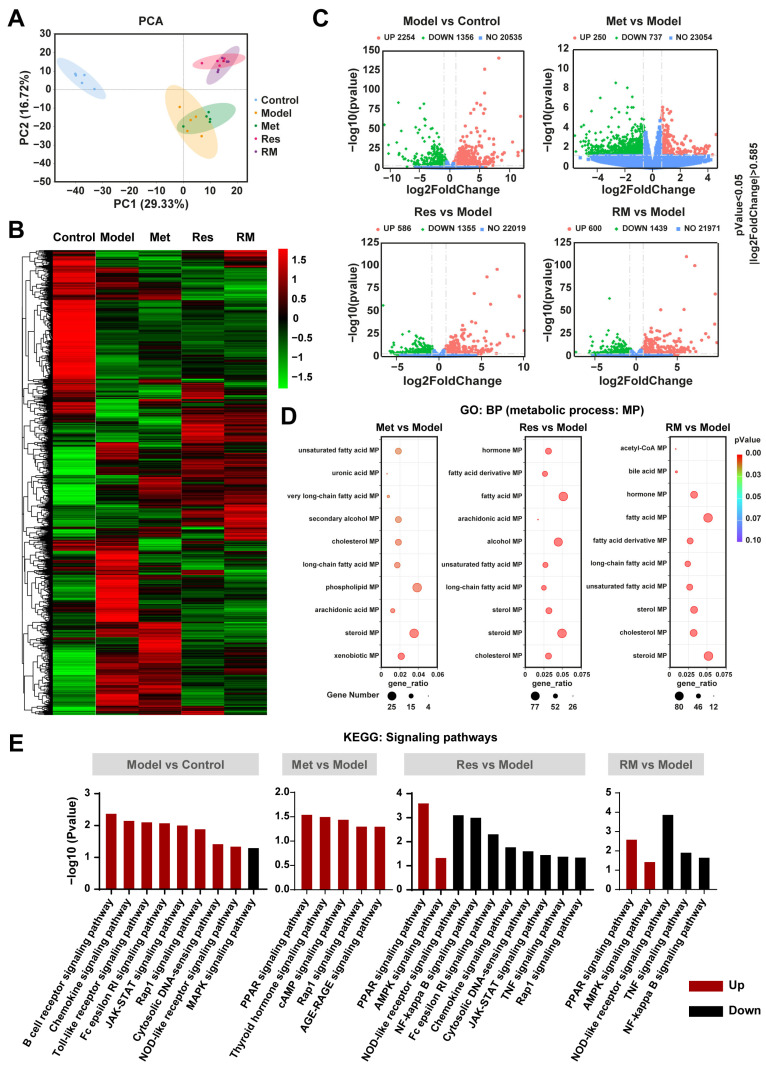
RM improved MASH in a similar way to Res based on transcriptomics analysis of mice liver tissue samples (*n* = 5/group). (**A**) PCA analysis of the control group, model group, Met group (410 mg/kg), Res group, and RM group. (**B**) Heatmap of RNA-sequencing among these five groups. (**C**) Volcano plots of upregulated and downregulated DEGs between two groups (*p* < 0.05 and |log2FoldChange| > 0.585). (**D**) Bubble diagrams of GO enrichment between two groups. (**E**) KEGG analysis of DEGs between two groups.

To further study the underlying molecular mechanisms of RM in relieving MASH, we performed RNA-sequencing analysis. The principal components analysis (PCA) plot revealed a clear separation between the model group and the control group, with the three treatment groups also distinctly separated from the model group (Figure 5A). Notably, the RM and Res groups exhibited no separation, indicating their similar molecular profiles (Figure 5A). This observation was further corroborated by the heatmap analysis, which showed highly similar gene expression profiles between the RM and Res groups (Figure 5B). Met, Res, and RM treatment significantly changed the gene expression profile in the liver of db/db mice, with a substantial number of differentially expressed genes (DEGs) undergoing both upregulation and downregulation (Figure 5C). Gene ontology (GO) enrichment analysis identified multiple biological processes (BP) that were related to lipid metabolic processes (MP), such as sterol MP, steroid MP, and cholesterol MP (Figure 5D). Subsequently, we conducted a Kyoto Encyclopedia of Genes and Genomes (KEGG) enrichment analysis. The data of signaling pathway enrichment indicated that several inflammation-related pathways were upregulated in the model mice compared to the control mice, while the MAPK signaling pathway was downregulated. Met was found to activate PPAR, thyroid hormone, cAMP, Rap1, and AGE-RAGE signaling pathways. Importantly, both Res and RM not only upregulated PPAR and AMPK signaling pathways but also downregulated NOD-like receptor, TNF, and NF-kappa B signaling pathways (Figure 5E). These findings imply that RM may attenuate MASH through mechanisms similar to those of Res, and all three administration methods remarkably modulate lipid metabolism processes.

### 3.6. Met, Res, and RM Extensively Changed the Lipid Profile in the Liver

**Figure 6:**
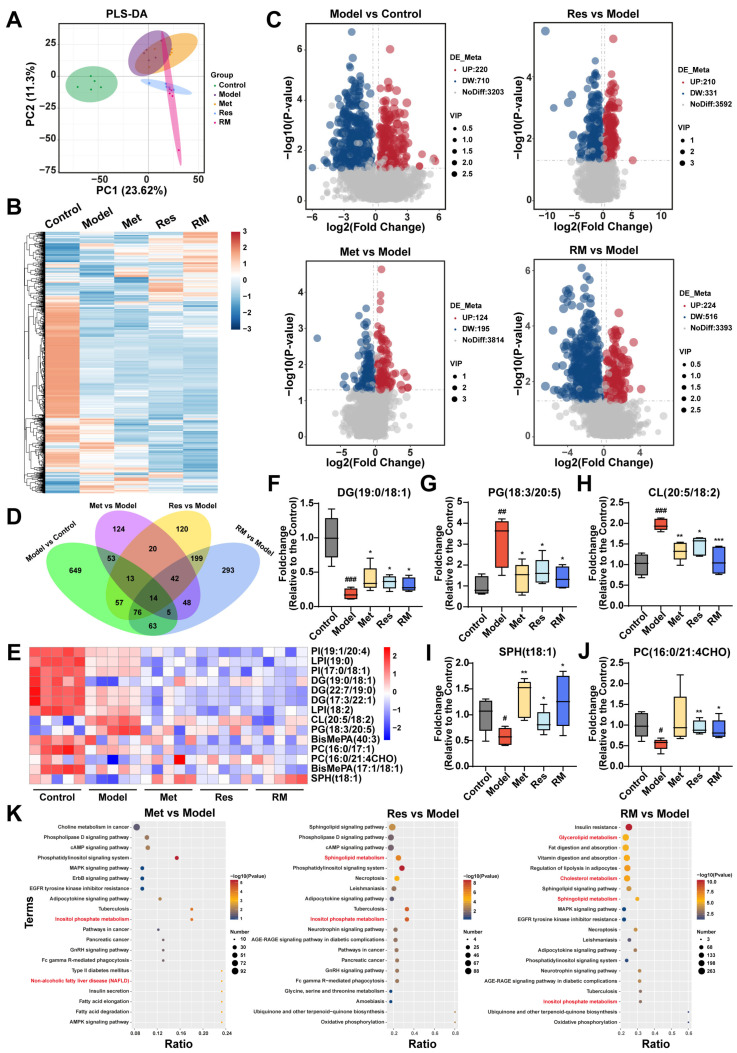
Met, Res, and RM improved liver lipid profiles of db/db mice (*n* = 5/group). (**A**) PLS-DA plot of the control group, model group, Met group (410 mg/kg), Res group, and RM group. (**B**) Heatmap of DALs among these five groups. (**C**) Volcano plots of upregulated and downregulated DALs between two groups (*p* < 0.05 and |log2FoldChange| > 0.263). (**D**) Venn plot of common DALs among four compare pairs. (**E**) Heatmap of 14 common DALs selected from four compare pairs. (**F**–**J**) Fold changes of five representative DALs selected from 14 common DALs. (**K**) KEGG analysis of DALs between two groups. Data represent mean ± SD; # *p* < 0.05, ## *p* < 0.01, and ### *p* < 0.001 versus the control group; * *p* < 0.05, ** *p* < 0.01, and *** *p* < 0.001 versus the model group.

To further explore the impact of Met, Res, and RM on lipid metabolism in MASH, we conducted a lipidomic analysis to examine the hepatic lipid profile transformations among control, model, Met (410 mg/kg), Res, and RM groups. Partial least squares discrimination analysis (PLS-DA) was employed to provide an overview of the separation degree among these five groups, suggesting distinct alterations in liver lipids between C57BL/6 mice and db/db mice (Figure 6A). Interestingly, the RM group showed a distant relationship with the model group but closer proximity to the Res group, consistent with the findings from transcriptomic analysis (Figure 5A and Figure 6A). Heatmap and volcano plots of differentially altered lipids (DALs) further demonstrated that the hepatic lipid profiles were significantly disrupted in db/db mice, while Met, Res, and RM treatments effectively adjusted the dysregulation of lipid metabolism (Figure 6B,C). By comparing DALs between the groups, 14 common DALs were screened from the control, model, and administration groups (Figure 6D). A heatmap was generated to illustrate the expression levels of these 14 DALs among the five groups (Figure 6E). Compared to control mice, the model mice exhibited significantly decreased levels of diradylglycerols (DG) (19:0/18:1), sphingoid bases (SPH) (t18:1), and glycerophosphocholines (PC) (16:0/21:4CHO) in their livers. Conversely, the levels of glycerophosphoglycerols (PG) (18:3/20:5) and glycerophosphoglycerophosphoglycerols (CL) (20:5/18:2) were notably increased in the model group. However, treatment with Met, Res, and RM markedly restored the levels of these lipids (Figure 6F–J).

Thereafter, KEGG analysis was further performed, as depicted in Figure 6K. DALs in Met, Res, and RM groups compared to the model were significantly enriched in terms related to lipid metabolism, such as inositol phosphate metabolism, sphingolipid metabolism, cholesterol metabolism, and glycerolipid metabolism (Figure 6K). These observations collectively suggest that Met, Res, and RM effectively modulate the intrahepatic lipid profiles in db/db mice, thereby potentially ameliorating lipid metabolic disturbances associated with MASH.

### 3.7. Comprehensive Analysis of Transcriptomics and Lipidomics in db/db Mice Treated with RM

**Figure 7:**
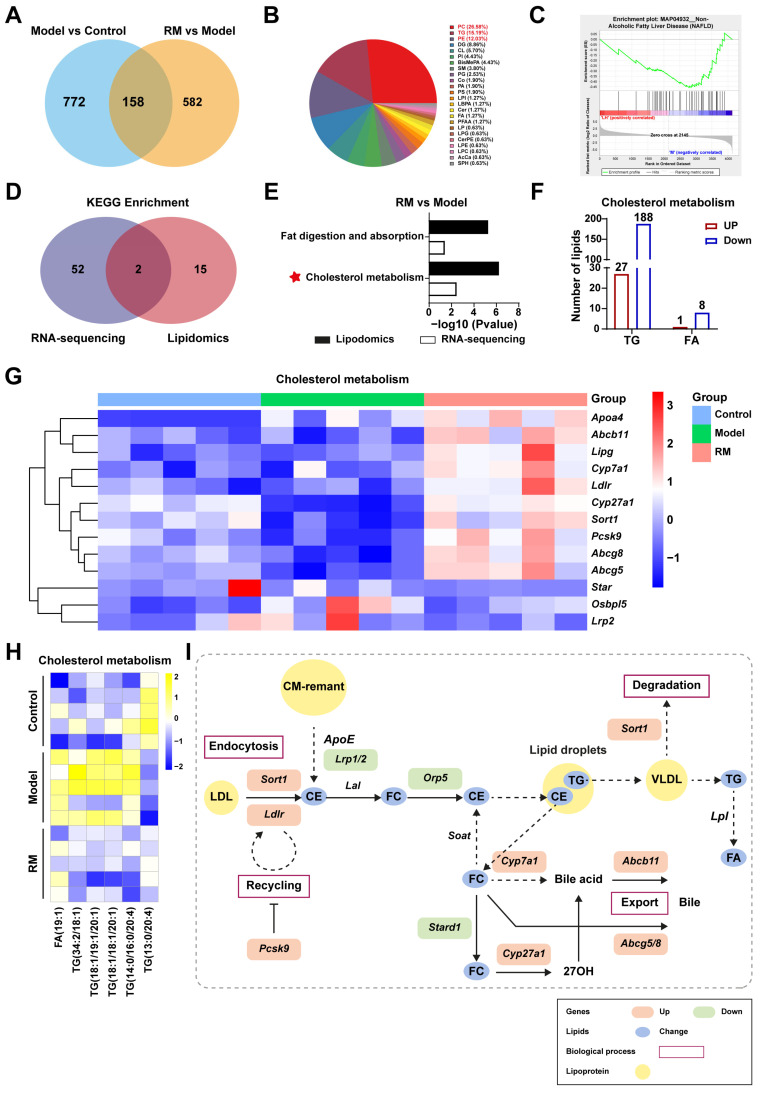
Comprehensive analysis of the transcriptomics and lipidomics in MASH mice treated with RM (*n* = 5/group). (**A**) Venn plot of common DALs among the control, model, and RM groups. (**B**) Pie chart of 158 common DALs selected from two compare pairs based on their lipid classes. (**C**) GSEA plot for KEGG entry: NAFLD based on DALs in the RM group vs. the model group. (**D**) Venn plot of the number of KEGG pathways among the control, model, and RM groups. (**E**) Bar plot of KEGG pathways among the control, model, and RM groups. (**F**) The number and type of DALs in the cholesterol metabolism pathway between the RM group and the model group. (**G**) Heatmap of gene expression profile related to cholesterol metabolism among the control, model, and RM groups. (**H**) Heatmap of six representative DALs selected from DALs related to cholesterol metabolism. (**I**) The biological process of cholesterol metabolism. The orange and green rectangles represented upregulated and downregulated genes after RM treatment, respectively. The blue dots represented altered lipids upon RM treatment. The yellow dots represented lipoproteins.

To further explore the specific mechanism of RM improving lipid metabolism in db/db mice, we conducted conjoint analysis of transcriptomics and lipidomics. RM treatment restored the levels of 158 DALs, as shown in Figure 7A. These DALs were further categorized into 24 distinct lipid classes and mainly concentrated in PC (26.58%), TG (15.19%), and glycerophosphoethanolamines (PE) (12.03%) (Figure 7B). Gene set enrichment analysis (GSEA) revealed that the downregulated lipids associated with NAFLD (KEGG entry: MAP04932) were significantly enriched in the RM group with an NES value of -1.579 and a *p*-value of 0.013 (Figure 7C). These results indicated that RM could impede MASH aggravation by modulating lipid homeostasis.

Next, we identified the overlapping pathways between the RM group and the model group through an integrated analysis of RNA sequencing and lipidomics, as illustrated in Figure 7D. RM was found to significantly impact pathways of fat digestion and absorption and cholesterol metabolism (Figure 7E). Dysregulation of cholesterol metabolism is a critical factor in MASH progression. Our data showed that RM increased the levels of 27 TGs and 1 FA, while decreasing the levels of 188 TGs and 8 FAs associated with cholesterol metabolism (Figure 7F). Disordered lipid levels were significantly corrected by RM (Figure 7H). Meanwhile, a heatmap was provided to better observe the changes of DEGs involved in cholesterol metabolic pathways (Figure 7G). Several key genes in lipoprotein uptake, cholesterol transformation, and bile excretion were displayed in Figure 7G, such as *Ldlr*, *Cyp7a1*, *Cyp27a1*, *Abcb11*, and *Abcg5/8*. Finally, an outline map was provided to intuitively illustrate the relationship between screened DALs and DEGs, which were significantly enriched in the livers of MASH mice after RM treatment (Figure 7I). Along with these findings, we refined the scope of our study to focus solely on cholesterol metabolism for subsequent validation.

### 3.8. RM Ameliorated MASH by Promoting Cholesterol Metabolism in the Fatty Liver of db/db Mice

**Figure 8:**
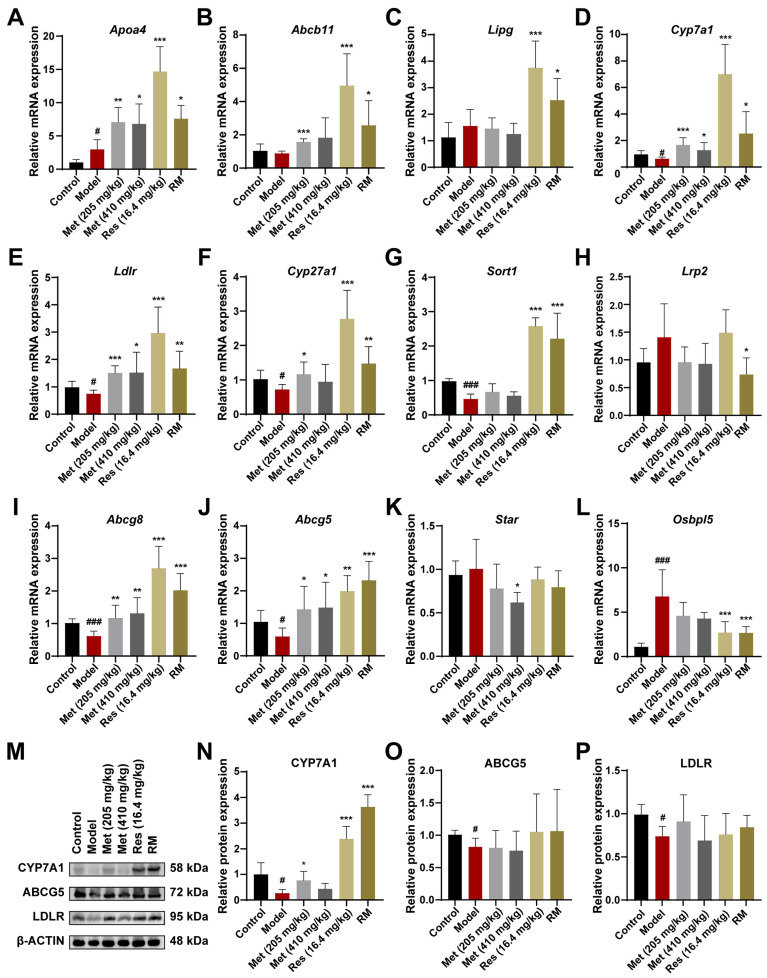
RM upregulates the cholesterol metabolism pathway by synergistically promoting the expression of CYP7A1 to prevent MASH. (**A**–**L**) The mRNA levels of DEGs enriched in the cholesterol metabolism pathway in mice liver were measured by RT-qPCR (*n* = 6). (**M**–**P**) The expression levels of CYP7A1, ABCG5, and LDLR in mice liver tissues were detected by Western blot experiment with β-ACTIN as the internal reference (*n* = 4). Data represent mean ± SD; # *p* < 0.05, ### *p* < 0.001 versus the control group; * *p* < 0.05, ** *p* < 0.01, and *** *p* < 0.001 versus the model group.

We further confirmed and compared the regulative roles of RM, Met, and Res in cholesterol metabolism at both the mRNA and protein levels. The expression patterns of the essential genes associated with cholesterol metabolism were consistent with the transcriptome data, which reinforced the reliability of our hypotheses (Figure 8A–L). Additionally, the levels of key enzymes in cholesterol metabolism were validated through Western blot experiments. The expression of CYP7A1, LDLR, and ABCG5 was significantly downregulated in the livers of model mice, suggesting that the biological processes of cholesterol clearance from plasma, cholesterol conversion to bile acids, and bile acid excretion in the liver were inhibited (Figure 8M–P). Intriguingly, CYP7A1 is a primary rate-limiting enzyme that catalyzes the transformation of cholesterol into bile acids [26]. Both Res and RM drastically promoted the expression of CYP7A1, with a stronger effect than Met, implying that CYP7A1 may be the potential target of Res (Figure 8M,N). These findings provide evidence that RM exerts its therapeutic effect on MASH through the activation of the cholesterol metabolism pathway.

## 4. Discussion

The incidence of MASLD continues to rise, and in its advanced stage, MASH is an important cause of cirrhosis and HCC, which seriously threatens human health [5]. However, currently approved treatments for MASH are limited, highlighting the urgent need to identify novel therapies. In this study, we established MASLD cell models by treating HepG2 and AML12 cells with FFA and used db/db mice as an animal model to evaluate the effects of Met, Res, and RM on MASH. Our results showed that all administration methods reduced lipid accumulation and alleviated lipid metabolism disorders in vitro. Meanwhile, lowered blood lipid levels, less liver lipid accumulation, inhibited inflammatory response, and improved liver function were observed in db/db mice after medication. Notably, through the comprehensive analysis of transcriptomics and lipidomics, we proved that the combination of half-dose Met and Res more effectively ameliorated inflammation and promoted cholesterol metabolism compared to Res alone.

Res, a selective THR-β agonist, has been approved for the treatment of non-cirrhotic MASH patients with moderate to advanced fibrosis [27]. It has been proven to effectively attenuate liver steatosis, inflammation, and fibrosis [28]. Additionally, Met has also been reported to improve liver steatosis in metabolic diseases through substantial preclinical studies [29,30,31]. However, the high cost of Res and the potential side effects of long-term high-dose medication still cannot be ignored. In the USA, Res treatment increased the cost by USD 66,764 per patient while increasing quality-adjusted life-years (QALYs) by 1.24 [32]. Therefore, combining halved doses of Res and Met may represent a novel therapeutic strategy, offering a lower economic burden for patients. Our results indicate that RM, Met (410 mg/kg), and Res exhibit comparable efficacy in MASH treatment.

HepG2 and AML12 cells, derived from human and mouse sources, respectively, are commonly used as cell models for MASLD [33,34]. In our experiments, FFA treatment significantly promoted the expression of lipid synthesis genes while inhibiting the expression of lipid catabolism genes. This led to increased levels of TG and TC, as well as enhanced lipid accumulation, findings that are consistent with previous reports [33,34,35]. These changes were significantly mitigated by treatment with RM, Met (40 μM), and Res. Other than in vitro observations, in vivo data showed that RM, Met (410 mg/kg), and Res notably alleviated balloon-like changes, lipid accumulation, inflammation, and fibrosis in the liver tissue of db/db mice. Concurrently, the levels of serum TG, TC, AST, and ALT were decreased, suggesting improvements in blood lipid profiles and liver function.

Furthermore, RNA sequencing was employed to unveil the potential mechanisms of RM, Met (410 mg/kg), and Res in MASH treatment. GO enrichment analysis revealed that multiple lipid metabolism processes were significantly altered in the treated groups, including sterol, steroid, long-chain fatty acid, and cholesterol metabolism. These findings were further corroborated by our lipidomic results. After supplementing with these three drugs, the lipid profile in the liver tissues of mice was markedly altered. Specifically, the levels of DG (19:0/18:1), SPH (t18:1), and PC (16:0/21:4CHO) were significantly increased, while the levels of PG (18:3/20:5) and CL (20:5/18:2) were notably decreased. DG (19:0/18:1) belongs to glyceride, which is involved in the last step of TG synthesis catalyzed by diacylglycerol acyltransferase 2 [36]. The medication increases DG content in liver tissues in this study, possibly caused by the inhibition of TG synthesis. PC (16:0/21:4CHO) is a phosphatidylcholine that reduces fat accumulation in the liver by promoting fat decomposition and transport, which helps to improve liver function and reduce liver damage [37]. The increase in PG (18:3/20:5) levels is typically associated with an imbalance between TG synthesis and excretion during the progression of MASLD. In line with our findings, Luukkonen et al. revealed that the mitochondrial amidoxime-reducing component 1 variant increases hepatic PCs and decreases the severity of MASLD in humans [38]. Collectively, these data demonstrate that RM, Met, and Res improve lipid metabolism during the development of MASH.

Notably, we studied the specific mechanisms by which RM modulates lipid metabolism. An integrated analysis of lipidomics and transcriptomics revealed that RM significantly affected cholesterol metabolism, fat digestion, and absorption. A plethora of evidence shows that cholesterol metabolism disorders deteriorate MASH [39,40,41]. The disorder of cholesterol metabolism in hepatocytes of MASLD mainly includes the activation of the cholesterol biosynthesis pathway and the inhibition of cholesterol export and bile acid synthesis [26]. LDLR is a crucial protein in the hepatocyte cholesterol metabolism pathway, mediating the endocytosis of LDL to maintain plasma LDL levels [42]. Cholesterol is converted into bile acids in the liver primarily through the actions of CYP7A1 and CYP27A1 [26]. Subsequently, bile acids are secreted from hepatocytes into the bile ducts, primarily via ABCG5/ABCG8, to complete the excretion process [43]. Our findings suggested that RM robustly upregulated the mRNA levels of LDLR, CYP7A1, CYP27A1, and ABCG5/8 in a manner similar to Res. Importantly, Res exhibited the most pronounced effect on CYP7A1 expression among all treatment groups. This implies that the combination of low-dose Res and Met enhances cholesterol metabolism by promoting the conversion of cholesterol to bile acids, potentially driven by the half-dose of Res.

During the progression of MASLD, excessive lipid accumulation in the liver leads to lipotoxicity in hepatocytes, triggering oxidative stress and cellular damage [44]. In response, hepatocytes secrete chemokines that recruit mononuclear-derived macrophages and activate Kupffer cells, further exacerbating liver inflammation and injury [45]. Notably, RM demonstrated superior efficacy compared to Met and Res alone in inhibiting the inflammatory response, as evidenced by ELISA and RT-qPCR results. In particular, RM significantly improved the levels of IL-10 in both circulation and liver tissues. Transcriptome analysis further provided evidence that the NOD-like receptor signaling pathway and NF-κB signaling pathway were significantly downregulated in the RM group. NOD-like receptors are a class of pattern recognition receptors that regulate the formation of inflammasomes and induce the production of IL-1β and IL-18, thereby participating in the inflammatory response [46]. In MASH, this signaling pathway is activated, driving an inflammatory cascade [47]. Our sequencing data indicated that RM treatment effectively inhibited this pathway. Additionally, recent studies have shown that auranofin reduces NF-κB and IκBα levels to inhibit the NF-κB signaling pathway in vitro [48]. Similarly, Jing et al. confirmed that RG-I pectin-like polysaccharide from *Rosa chinensis* repressed inflammation and fibrosis by targeting the NF-κB signaling pathway in MASH [49]. Collectively, our data highlight that RM plays a better role in lessening inflammation during MASH progression.

However, our research still has several limitations that require further investigation. While our study revealed that combined administration dramatically promoted the expression of CYP7A1, the underlying mechanisms by which this combination regulates cholesterol metabolism remain unclear. It is yet unknown if the enhanced expression of CYP7A1 is directly connected to the therapeutic effects of the combination treatment on MASH. Additionally, although the combined treatment outperformed Res and Met individually in mitigating inflammation, it remains unknown whether the promotion of cholesterol metabolism is a crucial link in its anti-inflammatory effects. Moreover, our transcriptome data indicated that RM modulates several signaling pathways beyond cholesterol metabolism, yet we have not conducted a comprehensive analysis of these pathways. Furthermore, while we have clarified that the combination of half-dose Met and Res improved MASH, the specific reasons why Met can supplement the pharmacological effects and mechanisms of Res remain to be elucidated. Our future research will focus on addressing these issues.

## 5. Conclusions

Collectively, our study found that a combination of half-dose resmetirom and metformin significantly ameliorated MASH, which was notably better than the effect of administration alone in improving inflammation. Especially, the underlying mechanism was unmasked by multi-omics combination analysis and subsequent validation. Combination therapy facilitated cholesterol metabolism in a similar way to resmetirom, especially promoting CYP7A1 expression to accelerate cholesterol transformation. Our findings confirm the hypothesis that combination therapy protects against MASH by promoting cholesterol metabolism and inhibiting inflammation, highlighting that combination therapy is a promising therapeutic strategy for MASH patients with lower economic burdens.

## Figures and Tables

**Table 1:** The sequences of primers used for RT-qPCR.

Gene	Forward Primer	Reverse Primer
*H-18S*	GTAACCCGTTGAACCCCATT	CCATCCAATCGGTAGTAGCG
*H-ACADL*	AGGGGATCTGTACTCCGCAG	CTCTGTCATTGCTATTGCACCA
*H-ACOX1*	ACTCGCAGCCAGCGTTATG	AGGGTCAGCGATGCCAAAC
*H-PPARA*	TTCGCAATCCATCGGCGAG	CCACAGGATAAGTCACCGAGG
*H-CPT1A*	TCCAGTTGGCTTATCGTGGTG	TCCAGAGTCCGATTGATTTTTGC
*H-SCD1*	GGGGGTGTGCTGACAACTTA	AGGCCCCTTTTTCTACCAGC
*H-ACC*	ACAGTGGACAGAATTGAGGG	AGTGGAGCTAGAATTGGACTTG
*H-FASN*	AAGGACCTGTCTAGGTTTGATGC	TGGCTTCATAGGTGACTTCCA
*M-18S*	GCCGTTCTTAGTTGGTGGAG	AACGCCACTTGTCCCTCTAA
*M-Acadl*	TTTCCTCGGAGCATGACATTTT	GCCAGCTTTTTCCCAGACCT
*M-Acox1*	GGAAGACTTCCAATCATGCGATAG	GACAACAAAGGCATGTAACCCG
*M-Ppara*	AACATCGAGTGTCGAATATGTGG	CCGAATAGTTCGCCGAAAGAA
*M-Pparg*	GGAAGACCACTCGCATTCCTT	GTAATCAGCAACCATTGGGTCA
*M-Acc*	ACAGTGGACAGAATTGAGGG	AGTGGAGCTAGAATTGGACTTG
*M-Fasn*	CAGAGCAGCCATGGAGGAG	TCCGTGACCATCTCCACAC
*M-Srebf1*	GGCACTAAGTGCCCTCAACCT	GCCACATAGATCTCTGCCAGTGT
*M-Cpt1a*	TGGCATCATCACTGGTGTGTT	GTCTAGGGTCCGATTGATCTTTG
*M-Tnfa*	GACGTGGAACTGGCAGAAGAG	TTGGTGGTTTGTGAGTGTGAG
*M-Il10*	GCTCTTACTGACTGGCATGAG	CGCAGCTCTAGGAGCATGTG
*M-Col1a1*	CATAAAGGGTCATCGTGGCT	TTGAGTCCGTCTTTGCCAG
*M-Col1a2*	AAGGATACAGTGGATTGCAGG	TCTACCATCTTTGCCAACGG
*M-Timp1*	CGAGACCACCTTATACCAGCG	ATGACTGGGGTGTAGGCGTA
*M-Acta2*	GTGAAGAGGAAGACAGCACAG	GCCCATTCCAACCATTACTCC
*M-Apoa4*	CCAATGTGGTGTGGGATTACTT	AGTGACATCCGTCTTCTGAAAC
*M-Abcb11*	TCTGACTCAGTGATTCTTCGCA	CCCATAAACATCAGCCAGTTGT
*M-Lipg*	ATGCGAAACACGGTTTTCCTG	GTAGCTGGTACTCCAGTGGG
*M-Cyp7a1*	GGGATTGCTGTGGTAGTGAGC	GGTATGGAATCAACCCGTTGTC
*M-Ldlr*	TGACTCAGACGAACAAGGCTG	ATCTAGGCAATCTCGGTCTCC
*M-Cyp27a1*	CCAGGCACAGGAGAGTACG	GGGCAAGTGCAGCACATAG
*M-Sort1*	CCCGGACTTCATCGCCAAG	AGGACGAGAATAACCCCAGTG
*M-Lrp2*	AAAATGGAAACGGGGTGACTT	GGCTGCATACATTGGGTTTTCA
*M-Abcg8*	CTGTGGAATGGGACTGTACTTC	GTTGGACTGACCACTGTAGGT
*M-Abcg5*	AGGGCCTCACATCAACAGAG	GCTGACGCTGTAGGACACAT
*M-Star*	ATGTTCCTCGCTACGTTCAAG	CCCAGTGCTCTCCAGTTGAG
*M*-*Osbpl5*	TTCTGGGCTGCGAAAATGAG	GTCAGATCCATGCATAGCCTG

## Data Availability

The data of this study are available from the corresponding author upon reasonable request.

## Abbreviations

The following abbreviations are used in this manuscript:

MASH	metabolic dysfunction-associated steatohepatitis
Res	resmetirom
Met	metformin
RM	half-dose Res and Met
MASLD	metabolic dysfunction-associated steatotic liver disease
T2DM	type 2 diabetes mellitus
FFA	free fatty acids
HCC	hepatocellular carcinoma
THR-β	thyroid hormone receptor-beta
HMGCR	3-hydroxy-3-methylglutaryl coenzyme A reductase
LDLR	low-density lipoprotein receptor
CYP7A1	cytochrome P450 family 7 subfamily A member 1
CYP27A1	cytochrome P450 family 27 subfamily A member 1
ABCG5/G8	ATP-binding cassette subfamily G member 5 or G member 8
TG	triglyceride
ACADL	long-chain acyl-coenzyme A dehydrogenase
ACOX1	acyl-coenzyme A oxidase 1
PPARA	proliferator-activated receptor α
CPT1A	carnitine palmitoyltransferase 1α
SCD1	stearoyl-CoA desaturase 1
ACC	acetyl-CoA carboxylase
FASN	fatty acid synthase
CCK8	cell counting kit-8
PPARG	proliferator-activated receptor γ
SREBF1	sterol regulatory element binding transcription factor 1
H&E	hematoxylin and eosin
TEM	transmission electron microscope
ALT	alanine aminotransferase
AST	aspartate aminotransferase
ELISA	enzyme-linked immunosorbent assay
TNF-α	tumor necrosis factor-alpha
IL	interleukin
COL1A1	collagen type I alpha 1
COL1A2	collagen type I alpha 2
TIMP1	tissue inhibitor of metal protease 1
ACTA2	alpha actin 2
PCA	principal components analysis
DEGs	differentially expressed genes
GO	gene ontology
BP	biological processes
MP	metabolic processes
KEGG	Kyoto encyclopedia of genes and genomes
PLS-DA	partial least squares discrimination analysis
DALs	differentially altered lipids
DG	diradylglycerols
SPH	sphingoid bases
PC	glycerophosphocholines
PG	glycerophosphoglycerols
CL	glycerophosphoglycerophosphoglycerols
PE	glycerophosphoethanolamines
GSEA	gene set enrichment analysis
QALYs	quality-adjusted life-years
SDS-PAGE	sodium dodecyl-sulfate polyacrylamide gel
